# Comparing direct anterior approach versus posterior approach or lateral approach in total hip arthroplasty: a systematic review and meta-analysis

**DOI:** 10.1007/s00590-023-03528-8

**Published:** 2023-04-03

**Authors:** James Jia Ming Ang, James Randolph Onggo, Christopher Michael Stokes, Anuruban Ambikaipalan

**Affiliations:** 1 Faculty of Medicine, Nursing and Health Sciences, Monash University, Wellington Rd, Clayton, Victoria, 3800 Australia; 2 Department of Orthopaedic Surgery, Box Hill Hospital, Victoria, Australia; 3 Department of Orthopaedic Surgery, Royal Melbourne Hospital, Victoria, Australia

**Keywords:** Direct anterior approach, Lateral approach, Posterior approach, Posterolateral approach, Total hip arthroplasty, Total hip replacement

## Abstract

**Background:**

There are several approaches to THA, and each has their respective advantages and disadvantages. Previous meta-analysis included non-randomised studies that introduce further heterogeneity and bias to the evidence presented. This meta-analysis aims to present level I evidence by comparing functional outcomes, peri-operative parameters and complications of direct anterior approach (DAA) versus posterior approach (PA) or lateral approach (LA) in THA.

**Patients and methods:**

A comprehensive multi-database search (PubMed, OVID Medline, EMBASE) was conducted from date of database inception to 1st December 2020. Data from randomised controlled trials comparing outcomes of DAA versus PA or LA in THA were extracted and analysed.

**Results:**

Twenty-four studies comprising 2010 patients were included in this meta-analysis*.* DAA has a longer operative time (MD = 17.38 min, 95%CI: 12.28, 22.47 min, *P* < 0.001) but a shorter length of stay compared to PA (MD = − 0.33 days, 95%CI: − 0.55, − 0.11 days, *P* = 0.003). There was no difference in operative time or length of stay when comparing DAA versus LA. DAA also had significantly better HHS than PA at 6 weeks (MD = 8.00, 95%CI: 5.85, 10.15, *P* < 0.001) and LA at 12 weeks (MD = 2.23, 95%CI: 0.31, 4.15, *P* = 0.02). There was no significant difference in risk of neurapraxia for DAA versus LA or in risk of dislocations, periprosthetic fractures or VTE between DAA and PA or DAA and LA.

**Conclusion:**

The DAA has better early functional outcomes with shorter mean length of stay but was associated with a longer operative time than PA. There was no difference in risk of dislocations, neurapraxias, periprosthetic fractures or VTE between approaches. Based on our results, choice of THA approach should ultimately be guided by surgeon experience, surgeon preference and patient factors.

**Level of evidence I:**

Meta-analysis of randomised controlled trials.

## Introduction

Total hip arthroplasty (THA) is a highly successful treatment for hip osteoarthritis, offering significant pain relief and improved quality of life by restoring function and mobility [1]. THA has shown excellent results over time, with 10-year survivorship exceeding 95% [2]. Annually, over one million THA is performed worldwide and is projected to reach two million by 2030 [1], attributed to the increasing life expectancy and prevalence of osteoarthritis.

There are several surgical approaches to THA, including posterior approach (PA), lateral approach (LA) and direct anterior approach (DAA), all of which have their respective advantages and disadvantages. PA involves splitting of gluteus maximus to access the hip joint posteriorly. PA allows for excellent exposure of both acetabulum and femur and avoids disruption of the hip abductors [3]. However, PA has been associated with an increased dislocation risk compared to LA or DAA [3–5], though this risk can be reduced with careful implant positioning and posterior soft tissue repair [6]. LA involves splitting of gluteus medius to access the hip joint anterolaterally. It has a lower risk of dislocation but is associated with superior gluteal nerve injury, heterotopic ossification and impaired abductor function [3]. DAA is unique with its inter-nervous and intermuscular plane between sartorius and tensor fascia latae, leading to increasing popularity as a THA approach [3]. Reported advantages include shorter hospital stay [7], earlier functional recovery [8] and lower dislocation risks [9]. Disadvantages include risk of lateral femoral cutaneous nerve (LFCN) injury [10], periprosthetic fractures [11] and the presence of a prolonged learning curve of 100 cases [12, 13].

There is ongoing debate with no clear consensus on the most optimal THA approach. Although several meta-analyses on this subject have previously been published, these meta-analyses had included non-randomised controlled trials (RCT) [4, 5, 8, 11, 14–17] which limit the quality of evidence presented since selection and recall bias cannot be excluded. Hence, an updated meta-analysis incorporating only RCTs would be of value to present the highest evidence level.

This meta-analysis aims to present level I evidence by evaluating and comparing 1. functional outcomes, 2. peri-operative parameters and 3. complications of DAA versus LA or PA in THA.

## Material and methods

### Literature search

This meta-analysis was performed according to the Preferred Reporting Items for Systematic reviews and Meta-Analyses (PRISMA) criteria. A comprehensive multi-database search (PubMed, OVID Medline, EMBASE) was conducted from date of database inception to 1st December 2020. The Medical Subject Headings and Boolean operators utilized were: [(‘Total hip arthroplasty’ OR ‘Total hip replacement’) AND (Approach)]. Results were subsequently filtered for RCTs. Identified articles and their corresponding references were reviewed and considered for inclusion according to the selection criteria.

### Selection criteria

All RCTs directly comparing outcomes of DAA versus LA or PA in THA were considered for inclusion. Non-English language studies, non-peer-reviewed studies, conference abstracts, unpublished manuscripts and studies not directly comparing outcomes between THA approaches were excluded. Two independent authors reviewed studies retrieved from the initial search and excluded irrelevant studies. Abstracts and titles of remaining articles were then screened against the inclusion criteria. Included articles were critically reviewed according to a pre-defined data extraction form. Differences in opinions were resolved by discussion between the first two authors.

### Data extraction

Extracted data parameters include details on study designs, publication year, patient numbers, basic demographics, peri-operative parameters, functional outcomes and complications. Peri-operative parameters include mean operative time (minutes), mean length of stay (LoS) (days), mean blood loss (millilitres), transfusion requirement, discharge destination and post-operative opioid use. Functional outcomes of interest include Harris Hip Score (HHS), Oxford Hip Score (OHS), Western Ontario and McMaster Universities Osteoarthritis Index score (WOMAC), EuroQoL 5-Dimension (EQ-5D), Hip Disability and Osteoarthritis Outcome Score (HOOS), Visual Analogue Scale (VAS) pain scores, 12-Item Short Form Health Survey (SF12), 36-Item Short Form Health Survey (SF36), University of California Los Angeles (UCLA) activity scores, Lower Extremity Functional Scale (LEFS) and timed up and go (TUG). Complications of interest include periprosthetic fractures, dislocations, venous thromboembolism (VTE), neurapraxia, wound dehiscence, superficial infections, deep infections and revisions. Data extracted were organised using a Microsoft Excel spreadsheet.

### Methodology assessment

Methodology quality of included studies was assessed with the Cochrane collaboration tool for Risk of Bias (RoB) in RCT [18]. Seven criteria were used to assess RCT, and each criterion was scored in three categories. The criterion is rated ‘low risk’ if the criterion is explicitly adhered to, ‘high risk’ if it is not adhered to and ‘unclear risk’ if the criterion is not mentioned. Any discrepancy in risk assessment was resolved by open discussion and a deciding vote from a third reviewer.

### Statistical analysis

Comparative meta-analysis was performed with odds ratio (OR) and weighted mean difference (MD) primarily used as summary statistics. In this meta-analysis, both fixed- and random-effects models were tested. Fixed-effects model assumed that treatment effects in each study were identical, while random-effects model assumed that variations were present between studies. *X*^2^ tests were used to study heterogeneity between studies. *I*^2^ statistic was used to estimate the percentage of total variation across studies, owing to heterogeneity rather than chance. Values greater than 50% were regarded as substantial heterogeneity. *I*^2^ can be calculated as: *I*^2^ = 100% x *(Q−df)/Q*. *Q* was defined as Cochrane’s heterogeneity statistics and *df* defined as degree of freedom. If substantial heterogeneity was present, the possible clinical and methodological reasons were explored qualitatively. This meta-analysis presented results with a random-effects model to account for clinical diversity and methodological variation between studies. All *p* values were two-sided. Review Manager (version 5.3, Copenhagen, The Nordic Cochrane Centre, The Cochrane Collaboration, 2014) were used for statistical analysis.

## Results

### Literature search

**Fig. 1:**
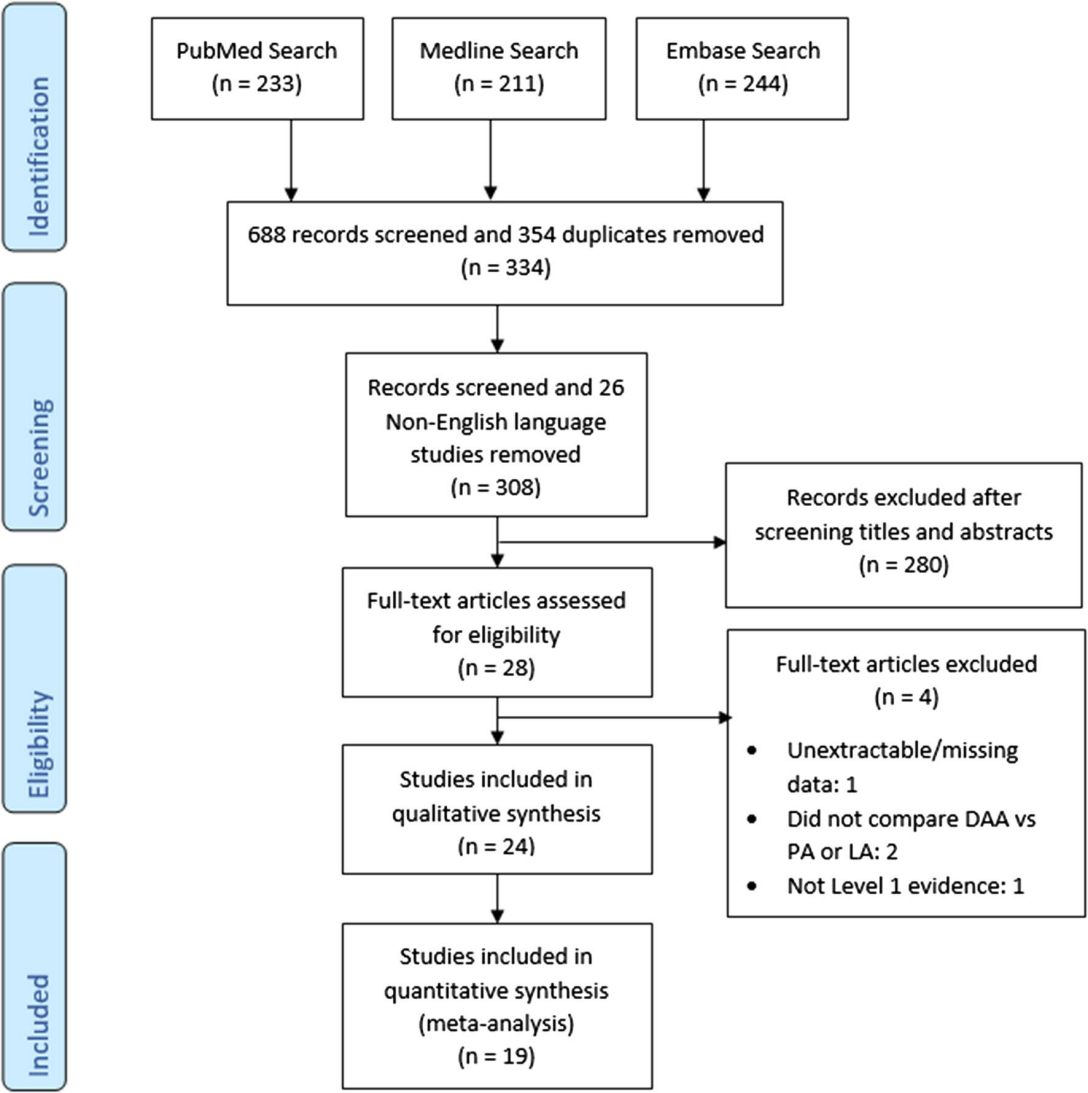
PRISMA search flowchart

A selection process flowchart to include relevant studies is illustrated in Fig. 1. A total of 688 studies were identified from initial search, of which 354 duplicates and 26 non-English language articles were removed. Titles and abstracts of 308 remaining studies were screened according to the pre-defined inclusion criteria, and 280 studies were excluded. Twenty-eight full-text articles were assessed for eligibility. Eventually, 24 randomized controlled trials were included of which 12 compared DAA versus PA [19–30] and 12 compared DAA versus LA [31–42].

### Methodology assessment

Risk of bias assessment summary and graph for all 24 included RCTs are found in Tables 1 and 2, respectively. Sixteen studies had low risk of bias in random sequence generation, while 8 studies had unclear risk. Risk of bias with allocation concealment was low in 11 studies but unclear in 13 studies. All studies had unclear or high risk of bias in blinding of participants and personnel due to nature of intervention. In terms of blinding of outcome assessors, two studies had high risk of bias, 13 had unclear risk, and 9 were low risk. Risk of bias with incomplete outcome data was low in 17 studies, unclear in five studies and high in two studies. Four studies had high risk of bias from selective reporting, while 20 were low risk. Apart from three studies with an unclear risk of other biases, the rest were of low risk.

**Table 1:**
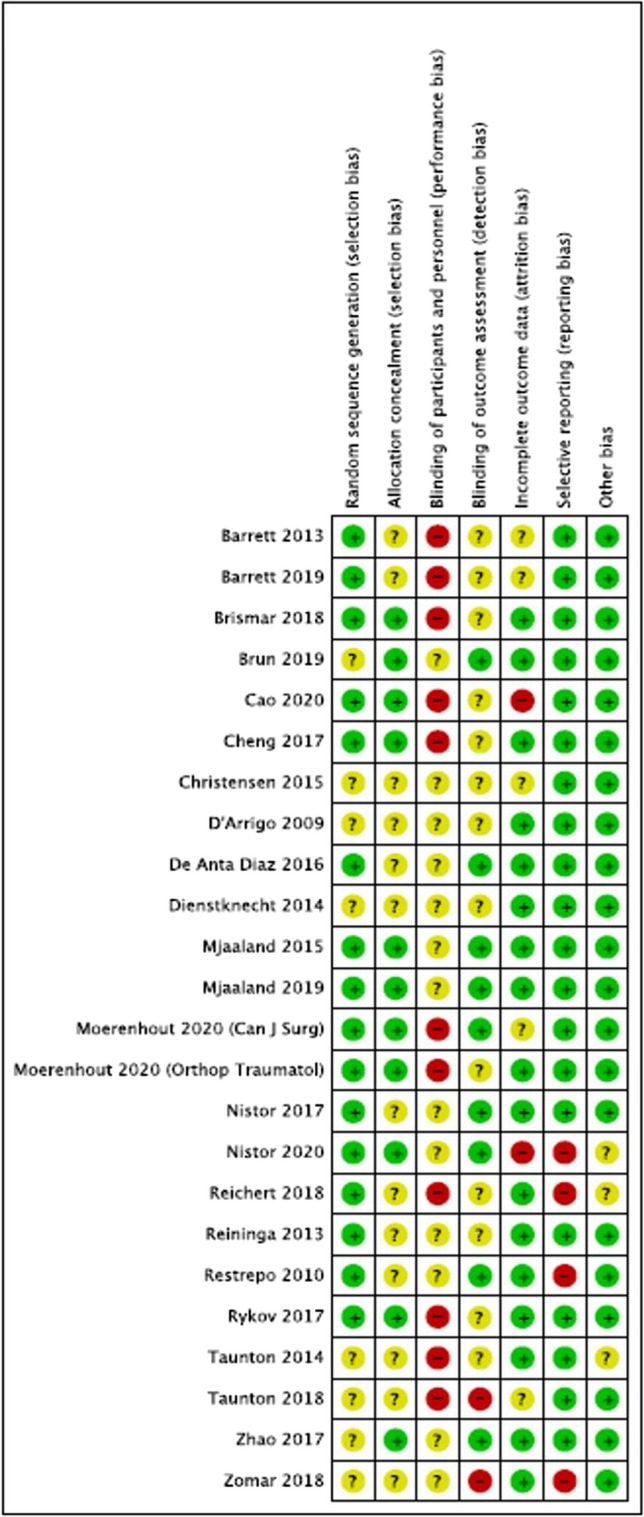
Risk of bias (RoB) assessment tool summary

**Table 2:**
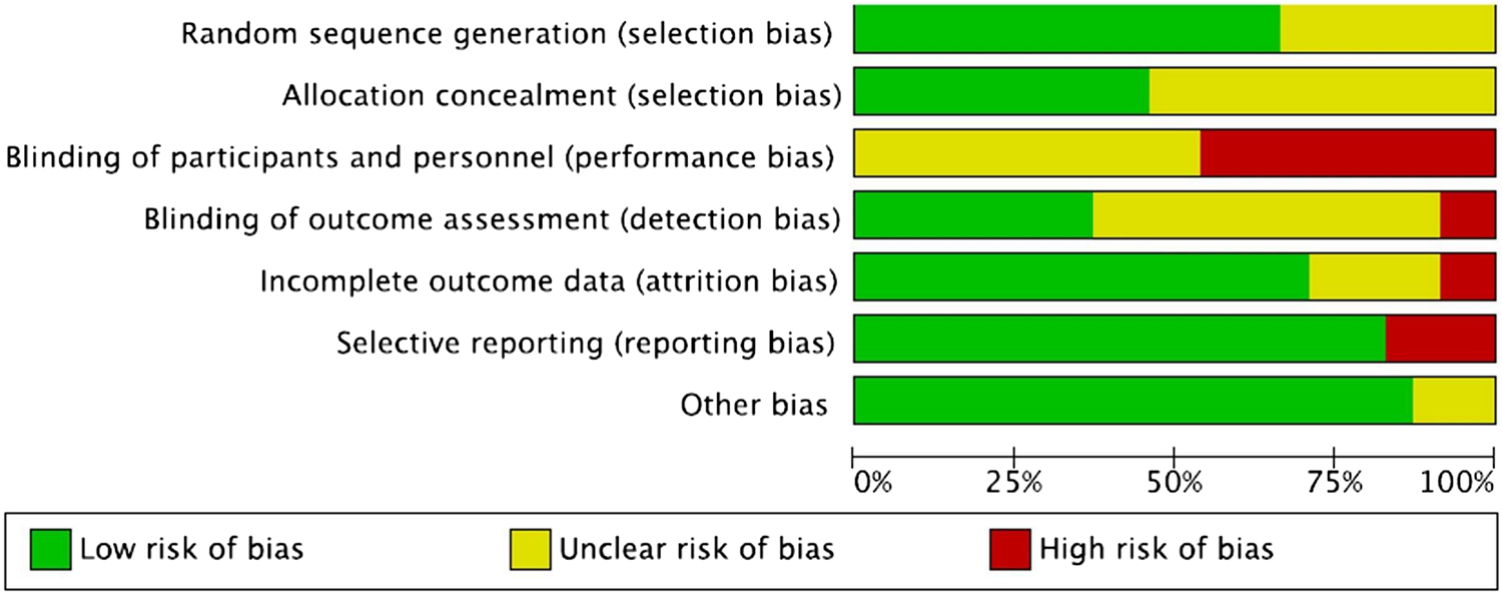
Risk of bias (RoB) assessment tool graph

### Demographics

A total of 2010 patients were included, with 792 in DAA versus PA and 1218 in DAA versus LA. Comparing DAA versus PA, both DAA and PA groups had 177 males and 219 females. Mean age in the DAA group was 63.5 years, while mean age of PA group was 63.3 years. Comparing DAA versus LA, 236 males and 361 females underwent DAA, while 288 males and 333 females underwent LA. Mean age was 64.7 years for the DAA group and 63.3 years for the LA group. Follow-up period was reported by 23 studies ranging from 4 days to 6.2 years. Other demographic details of each study are listed in Table 3.

**Table 3:** Basic demographics of included studies

Articles	Year	Study design	No of patients		Mean age		Sex				Follow-up in years (range)	
DAA vs PA			DAA	PA	DAA	PA	DAA		PA		DAA	PA
							Male	Female	Male	Female		
Barrett	2013	RCT	43	44	61.4	63.2	29	14	19	25	Up to 1	
Barrett	2019	RCT	43	44	61.4	63.2	29	14	19	25	4.94	5.19
Cao	2020	RCT	65	65	61.4	62.4	27	38	28	37	Up to 0.5	
Cheng	2017	RCT	35	38	59.0*	62.5*	15	20	18	20	Up to 0.25	
Christensen	2015	RCT	28	23	64.3	65.2	13	15	11	12	Up to 0.115	
Moerenhout (Can J Surg)	2020	RCT	28	27	70.4	68.9	11	17	18	9	4.583	
Moerenhout (Orthopaedics and traumatology)	2021	RCT	24	21	70.3	67.7	11	13	14	7	5.167 (4–6.167)	
Reininga	2013	RCT	35	40	60.3	60.5	11	24	8	32	Up to 0.5	
Rykov	2017	RCT	23	23	62.8	60.2	8	15	11	12	Up to 0.115	
Taunton	2014	RCT	27	27	62.1	66.4	12	15	13	14	1	
Taunton	2018	RCT	52	49	65.0	64.0	27	25	25	24	1.718	
Zhao	2017	RCT	60	60	64.9	62.2	24	36	26	34	Up to 0.5	

DAA vs LA			DAA	LA	DAA	LA	DAA		LA		DAA	LA
							Male	Female	Male	Female		
Brismar	2018	RCT	50	50	66*	67*	18	32	17	33	Up to 5	
Brun	2019	RCT	84	80	67.2	65.6	25	59	30	50	–	
D' Arrigo	2009	RCT	20	20	64.0	66.3	12	8	14	6	Up to 0.115	
De Anta Diaz	2016	RCT	50	49	64.8	63.5	26	24	26	23	1	
Dienstknecht	2014	RCT	55	88	61.9	61.3	22	33	41	47	0.25	
Mjaaland	2015	RCT	84	80	67.2	65.6	25	59	30	50	Up to 0.0110	
Mjaaland	2019	RCT	84	80	67.2	65.6	25	59	30	50	Up to 2	
Nistor	2017	RCT	35	35	67.0*	64.0*	9	26	19	16	0.25	
Nistor	2020	RCT	56	56	65.0*	63.0*	16	40	30	26	Up to 0.25	
Reichert	2018	RCT	77	71	63.2	61.9	45	32	39	32	Up to 1	
Restrepo	2010	RCT	50	50	62.0	59.9	17	33	22	28	2	
Zomar	2018	RCT	36	42	60.8	59.5	21	15	20	22	Up to 0.25	

* Values presented in median, '–' Data not available

### Clinical outcomes

**Fig. 2:**
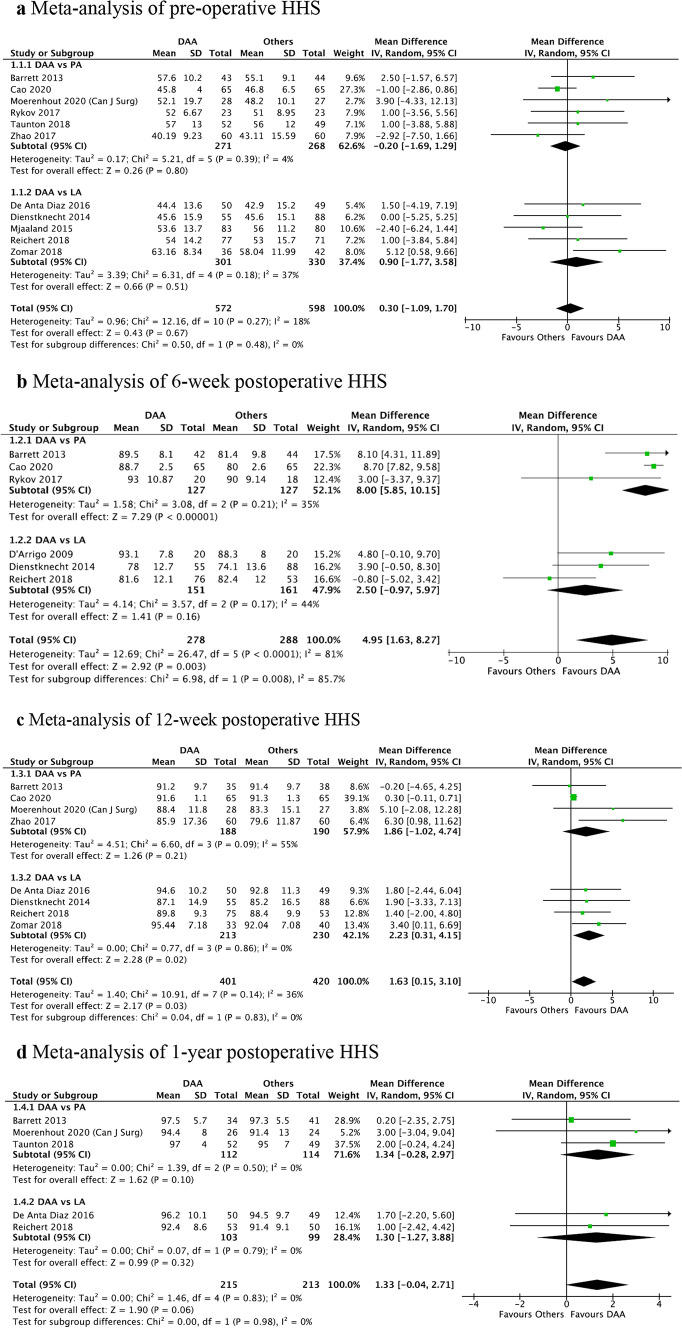
**a** Meta-analysis of pre-operative HHS, **b** meta-analysis of 6-week post-operative HHS, **c** meta-analysis of 12-week post-operative HHS, **d** meta-analysis of 1-year post-operative HHS

Comparing DAA versus PA, there was a significantly better HHS in the DAA than PA group at 6 weeks (mean difference (MD) = 8.00, 95%CI: 5.85, 10.15, *P* < 0.001) as seen in Fig. 2b, while pre-op (MD = − 0.20, 95%CI: − 1.69, 1.29, *P* = 0.80), 12 week (MD = 1.86, 95%CI: − 1.02, 4.74, *P* = 0.21) and 1-year (MD = 1.34, 95%CI: − 0.28, 2.97, *P* = 0.10) HHS did not show statistically significant difference (Fig. 2b–d).

When comparing DAA versus LA, there was a significantly better HHS in the DAA than LA group at 12 weeks (MD = 2.23, 95%CI: 0.31, 4.15, *P* = 0.02) as seen in Fig. 2c, while pre-op (MD = 0.90, 95%CI: − 1.77, 3.58, *P* = 0.51), 6 week (MD = 2.50, 95%CI: − 0.97, 5.97, *P* = 0.16) and 1-year (MD = 1.30, 95%CI: − 1.27, 3.88, *P* = 0.32) HHS did not show statistically significant difference (Figs. 2a, b, d).

Due to heterogeneity of PROMS, comparative statistical analysis could only be performed for pre-op, 6-week, 12-week and 1-year HHS. All other functional outcomes are summarised in Appendix 1.

Eleven RCTs discussed pain scores. Seven RCTs reported lower VAS pain scores in the first few days up to 1-week post-operatively for DAA [24, 25, 28, 31, 35, 36, 38]. Four studies noted no significant difference beyond 2 weeks [19, 22, 25, 37]. Cao et al. [27], however, reported lower pain scores for DAA at 3 and 6-weeks when comparing DAA versus PA.

In terms of gait parameters, there were inconsistent results across studies. Comparing DAA versus PA, Zhao et al. reported improved gait recovery at 3 months but not 6 months for DAA, while Reininga et al. [28, 30] reported no difference in locomotor parameters and gait recovery, respectively. Comparing DAA versus LA, Zomar et al. [42] found improved gait velocity, stride length, step length and symmetry at early follow-up favouring DAA.

### Radiological

Nine RCTs discussed radiological positioning. Eight RCTs reported no significant difference in radiological positioning of implants between THA approaches [19, 21–23, 32, 35, 38, 40]. However, Zhao et al. [28] concluded that the DAA was associated with more accurate cup positioning.

### Peri-operative parameters

**Fig. 3:**
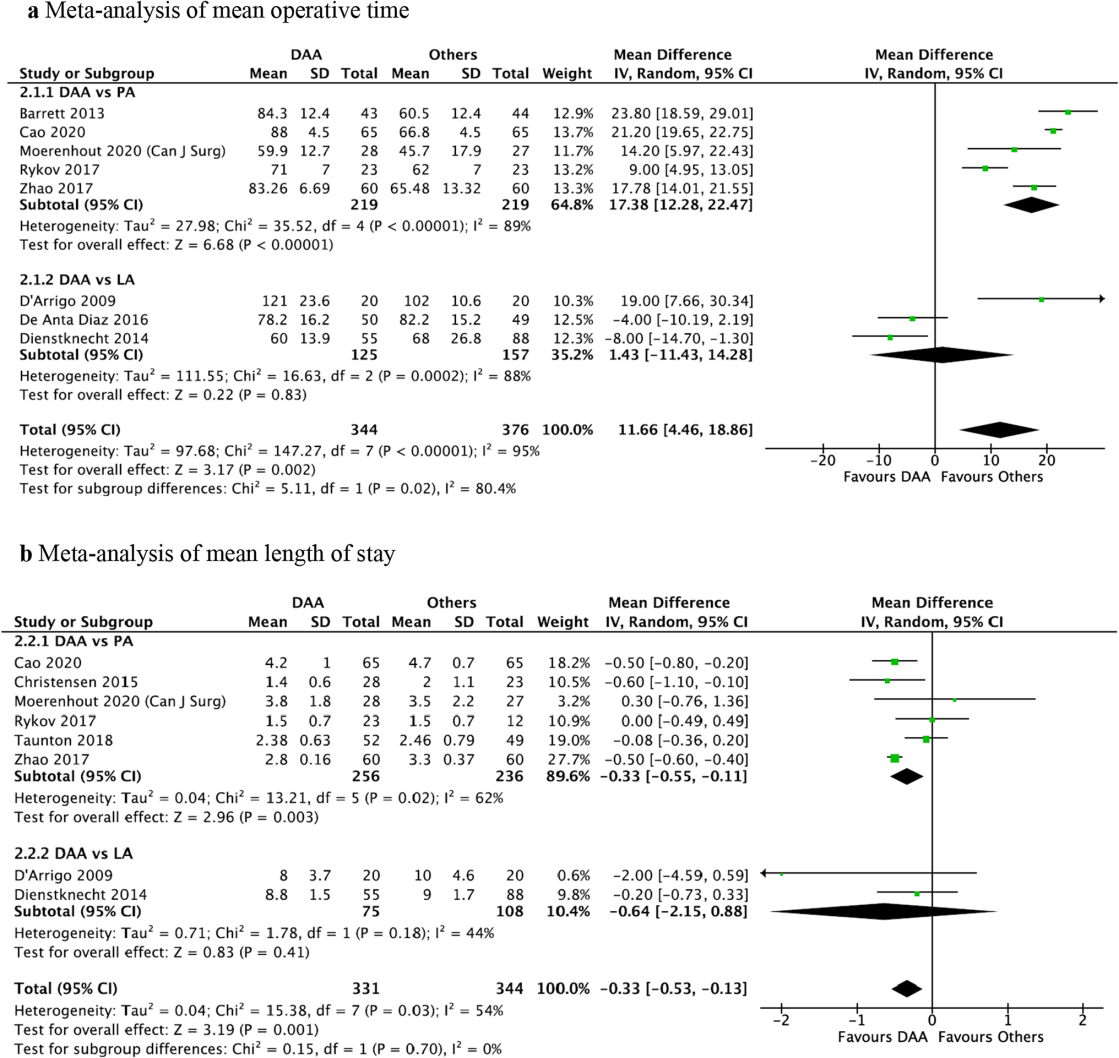
**a** Meta-analysis of mean operative time, **b** meta-analysis of mean length of stay

Mean operative time was significantly longer for DAA compared to PA (MD = 17.38 min, 95%CI: 12.28, 22.47 min, *P* < 0.001), but there was no significant difference between DAA and LA (MD = 1.43 min, 95%CI: − 11.43, 14.28 min, *P* = 0.83) (Fig. 3a).

Mean LoS was significantly shorter for DAA versus PA (MD = -0.33 days, 95%CI: − 0.55, − 0.11 days, *P* = 0.003), but there was no statistically significant difference between DAA and LA (MD = − 0.64 days, 95%CI: − 2.15, 0.88 days, *P* = 0.41) (Fig. 3b).

No statistical analysis could be performed for other peri-operative parameters due to heterogeneity of raw data. Four studies comparing DAA versus PA noted higher blood loss in DAA [19, 25, 27, 28], while seven studies comparing DAA versus LA did not report any significant difference [31, 33, 35, 36, 38, 41]. Several studies also reported significantly lower morphine equivalents required in DAA patients post-operatively [19, 24, 31, 36, 38], while others did not [25, 41]. Studies that evaluated transfusion rates [19, 27, 28, 36, 38, 41] and discharge destination [19, 41] did not notice any difference between DAA and other approaches.

### Complications

**Fig. 4:**
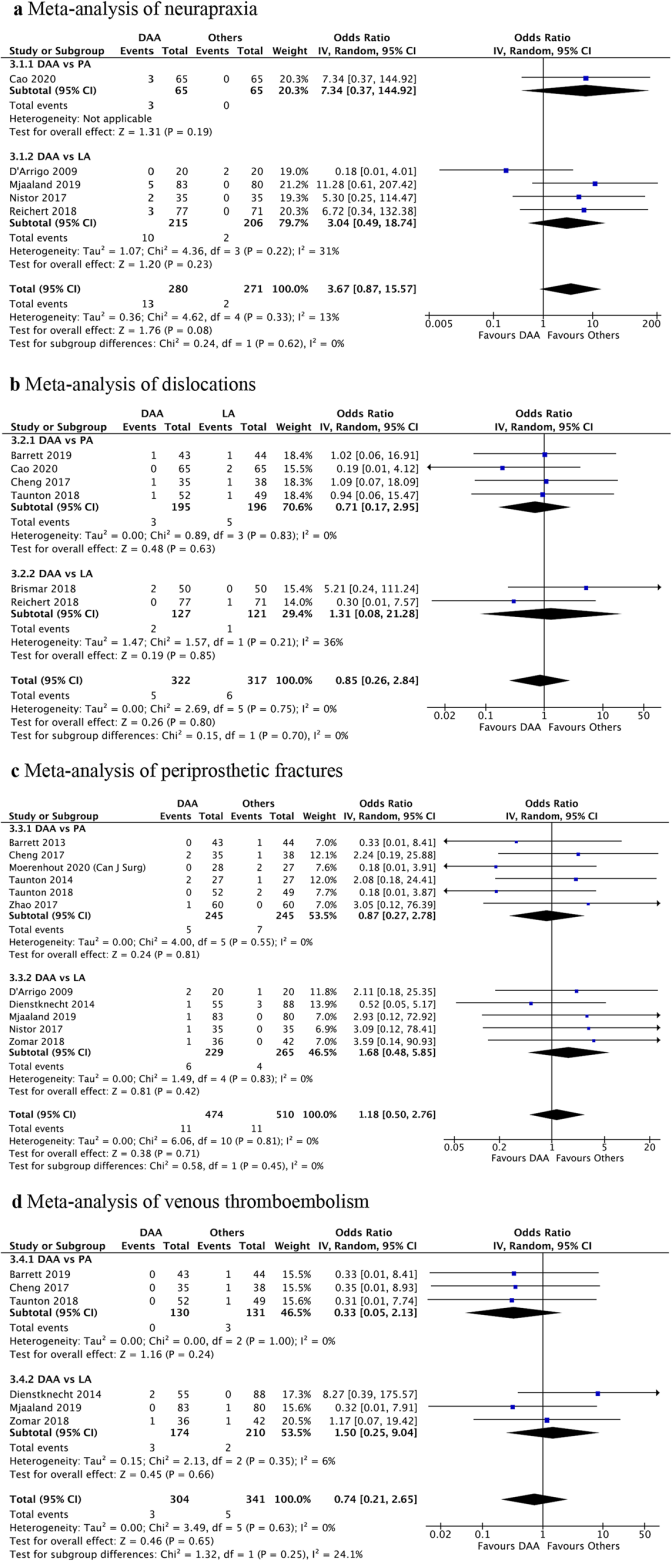
**a** Meta-analysis of neurapraxia, **b** meta-analysis of dislocations, **c** meta-analysis of periprosthetic fractures, **d** meta-analysis of venous thromboembolism

There was no significant difference in risk of neurapraxia between DAA and LA (OR = 3.04, 95%CI: 0.49, 18.74, *P* = 0.23). Meta-analysis for neurapraxia risk for DAA versus PA could not be performed as only Cao et al. reported neurapraxia rates [27] (Fig. 4a). Otherwise, there was no statistically significant difference in risk of dislocations, periprosthetic fractures or venous thromboembolisms when comparing DAA versus PA or LA (Figs. 4b–d).

## Discussion

This is an updated comprehensive level-1 meta-analysis comparing functional outcomes, peri-operative parameters and complications of THA performed via DAA versus PA or LA. Most prominently, DAA had better functional outcomes in terms of HHS in the early post-operative period, with statistically significant difference at 6 weeks over PA and at 12 weeks over LA. While DAA had a slightly shorter mean length of stay than PA, DAA was associated with a significantly longer operative time than PA. There was no difference in risk of neurapraxia for DAA vs LA, and there was no difference in risks of dislocations, periprosthetic fractures or VTE between approaches.

An updated meta-analysis is justified due to increasing numbers of new RCTs published on this topic. The strict inclusion of only RCTs ensures that biases are minimised to produce the highest evidence level. While previous meta-analyses mainly compared two surgical approaches, our meta-analysis compared three main surgical approaches currently valid in clinical practice, with DAA being the common comparison. A network meta-analysis was not performed since assumptions associated with performing the analysis would reduce quality of evidence. Instead, our meta-analysis presents subgroup analysis comparing DAA with PA or LA and an overall analysis comparing DAA with PA and LA. This allows for direct comparison between DAA and other common approaches without compromising quality of evidence as with network meta-analysis.

DAA showed earlier recovery of function in the early post-operative period, which is consistent with previously published meta-analyses [5, 8, 11, 14, 17]. The quicker recovery has been attributed to the muscle-sparing nature of DAA by utilizing an inter-nervous plane between tensor fasciae latae and sartorius muscle superficially and between gluteus medius and rectus femoris deeper. Hence, muscle splitting is avoided and soft tissue injury is minimised [8, 43]. This is supported by biochemical and radiological evidence, with reports of lower levels of early post-operative creatine kinase or myoglobin, which are indicators of muscle damage, in DAA compared to other approaches [28, 34, 38, 39]. Post-operative MRI studies also noted less muscle and tendon damage in DAA than LA [34].

While no statistical analysis was performed for VAS pain scores, 8 of 11 RCTs reported lower levels of clinical pain measured by VAS in DAA versus other approaches. This could be attributed to minimal soft tissue trauma leading to earlier functional recovery. Pain is associated with poorer recovery following THA [44]. Progress of early post-operative rehabilitation is often limited and delayed due to pain; hence, lower pain VAS may be a positive driver and motivator of earlier rehabilitation. It should be noted that VAS pain levels and opioid requirements were only discussed qualitatively due to parameter heterogeneity. Post-operative analgesia regimes play a significant role in post-operative pain management, with the type of local anaesthetic used before skin closure, mode and type of analgesia used post-operatively greatly influencing VAS pain levels. Since analgesia regimes are not standardised across studies, it would be difficult to directly compare VAS pain without introducing bias.

HHS is a comprehensive instrument widely used to assess THA outcomes, comprising domains for pain severity, function, absence of deformity and range of motion. A study by Söderman et al. [45] concluded that HHS is a valid, reproducible and reliable indicator of clinical outcome after THA. The minimum clinically important difference (MCID) for HHS was reported to be 4 [46]. According to this measure, our results demonstrate a clinically significant improvement in HHS at 6 weeks for DAA versus PA but not at 12 weeks for DAA versus LA.

Previous meta-analyses comparing mean LoS in DAA versus PA have been inconsistent, with some reporting shorter LoS in DAA [5, 11], while others reporting no difference [8, 14]. Our study showed a slightly shorter LoS in DAA than PA, likely due to less soft tissue trauma in DAA and lower post-operative pain levels, which facilitates better tolerance and participation in early post-operative rehabilitation. Inconsistent results have also been reported for operative time between THA approaches, with some reporting increased operative time for DAA [11, 14], while others find no significant difference [5, 8]. This meta-analysis reports a longer operative time for DAA than PA postulated to be due to surgeon experience, the use of a fracture table and/or intraoperative fluoroscopy during DAA THA [25, 29]. Four RCTs noted higher blood loss for DAA versus PA. This could be attributed to the longer operative time for DAA over PA since blood loss has been noted to increase with surgical duration [47]. The long learning curve for DAA, which has previously been described, could be another contributing factor, though all but two [23, 28] of the RCTs comparing DAA versus PA involved surgeons experienced in DAA. While our results did not show any difference in peri-operative parameters between DAA and LA, Yue et al. [17] reported a longer operative time and shorter LoS for DAA compared to LA.

Overall, 14 of 24 RCTs involved surgeons experienced in DAA, [19–22, 24–27, 29, 30, 32, 35, 40, 42]. The remainder either involved surgeons still within the learning curve [28, 31, 33, 36–38, 41] or did not specify surgeon experience [23, 34, 39]. Complication risks during the learning curve of DAA can potentially be reduced with adequate supervision and guidance by experienced surgeons and by performing initial cases on less complex patients [48].

Although our study did not find an increased risk of neurapraxia for DAA vs LA and could not run the meta-analysis for DAA vs PA, previous meta-analyses have reported an increased risk of neurapraxia with DAA [11, 15, 16]. The LFCN is most often implicated in DAA as it lies within the intermuscular interval used for DAA with an incidence of 14.8–81% [49]. As a sensory nerve, the symptoms include numbness and neuropathic pain. LFCN injuries generally improve over time with several studies showing symptom improvement in over 88% of patients after 2 years [49]. On the other hand, the sciatic nerve is more likely to be implicated in the PA due to its posterior location. Although overall incidence of sciatic nerve injury is relatively low at 0.068–1.9% [49], the rate of full recovery is reportedly less than 50% [50]. Being a major motor nerve that supplies most of the posterior compartment musculature in the lower limb, an injury to the sciatic nerve can lead to debilitating functional consequences.

There was no significant difference in risk of dislocations, periprosthetic fractures or VTE between approaches, which is also consistent with previous meta-analysis [4, 8, 11, 15–17]. However, three meta-analyses did report a higher risk of dislocations in PA than DAA [4, 5, 15]. Medium-term data from the Australian Orthopaedic Association National Joint Replacement Registry (AOANJRR) also reported an increased risk of revision surgery in PA THA indicated for recurrent dislocations (HR = 1.84, 95%CI: 1.55, 2.20, *p* < 0.001). There are several reasons that could have led to this discrepancy in dislocation rates between our analysis and other reports. Firstly, including only RCTs meant that patient numbers are limited and there may be insufficient statistical power to demonstrate a significant difference. Furthermore, a majority of RCTs focused mainly on the early post-operative period which could be too early for all dislocations to occur. It was also noted that most PA THA included in this analysis was reported to have posterior capsule repair and/or peri-operative hip precautions to minimise the risk of dislocations. Other confounding factors for this discrepancy can be due to the higher numbers of PA for THA, differing indications for PA THA, differing soft tissue closure techniques and individual patient factors including soft tissue integrity and comorbidities.

### Limitations

There are several limitations to this meta-analysis. Due to heterogeneity of reported PROMS and their follow-up intervals, only comparative analysis of HHS could be performed. PROMS that could not be quantitatively analysed are summarised in Appendix 1 for easy comparison between surgical approaches. The difference in surgeon experience amongst studies is a potential confounder given the learning curve of DAA of 100 procedures, with an increased risk of complications if this minimum threshold is not met [12, 13]. Although complication rates compared were consistently low across studies, the wide difference in follow-up duration across studies could have impacted the number and type of complications observed. Hence, it would be difficult to account for the impact that the learning curve has on complications in this context. Unfortunately, we could not control or adjust for the influence that this discrepancy could have had on our results. Several RCTs reported utilising minimally invasive surgery (MIS) techniques to perform THA. To date, the definition of MIS remains debatable [51, 52]. Traditionally, it is perceived that MIS involves smaller incisions. However, studies have shown that there are more factors to MIS than incision length alone, with minimal soft tissue trauma being a key principle [51, 52]. Hence, it would be exceptionally challenging to adjust for this factor given the lack of a standardised definition of MIS. Although osteoarthritis was the main indication for a majority of THAs performed, the inclusion of other diagnoses may act as confounding variables. Detection bias may have been introduced considering that discharge criteria and blinding of outcome assessors were not clearly defined in some RCTs [27, 29]. Lastly, the quality of RCTs included was limited by the inherent inability to completely blind participants and researchers given the nature of the intervention.

## Conclusion

The DAA has better early functional outcomes with shorter mean length of stay and was associated with a longer operative time than PA. There was no difference in risk of neurapraxia for DAA vs LA, and there was no difference in risks of dislocations, periprosthetic fractures or VTE between approaches. Based on our results, preference of THA approach should ultimately be guided by surgeon experience, surgeon preference and patient factors.

## Appendix 1

See Table 4.

**Table 4:** Patient-reported outcome measures between approaches

Articles	Year	No of patients		Outcome measure	Mean (Standard deviation)		*P* value
DAA vs PA		DAA	PA		DAA	PA	
Barrett	2013	43	44	Pre-op VAS	4.8 ± 2.5	5.5 ± 2.3	0.1751
		43	44	Post-op immediate VAS	4.2 ± 1.4	4.6 ± 1.8	0.2257
		43	44	Day 1 VAS	4.0 ± 1.0	4.5 ± 1.2	**0.0472**
		43	44	Day 2 VAS	3.8 ± 1.1	4.1 ± 1.0	0.2042
		42	44	6-week VAS	1.9 ± 1.2	1.9 ± 1.6	0.953
		35	38	3-month VAS	1.3 ± 0.5	1.4 ± 1.0	0.4414
		34	36	6-month VAS	1.6 ± 1.5	1.4 ± 1.2	0.4606
		34	41	1-year VAS	1.6 ± 1.4	1.3 ± 0.6	0.1857
		43	44	Pre-op HHS, pain	17.3 ± 6.4	14.5 ± 5.0	**0.0347**
		42	44	6-week HHS, pain	39.8 ± 4.4	38.4 ± 5.4	0.2056
		35	38	3-month HHS, pain	37.5 ± 7.0	39.4 ± 6.2	0.2402
		34	36	6-month HHS, pain	41.1 ± 5.9	41.1 ± 5.7	0.9701
		34	41	1-year HHS, pain	42.0 ± 5.2	42.5 ± 4.4	0.6615
		43	44	Pre-op HHS, function	22.2 ± 5.0	22.4 ± 4.8	0.8685
		42	44	6-week HHS, function	28.7 ± 3.7	25.5 ± 5.3	**0.0027**
		35	38	3-month HHS, function	31.5 ± 2.8	30.6 ± 3.5	0.2371
		34	36	6-month HHS, function	32.4 ± 1.4	32.6 ± 1.3	0.6626
		34	41	1-year HHS, function	32.8 ± 0.7	32.4 ± 1.6	0.1301
		43	44	Pre-op HHS, total	57.6 ± 10.2	55.1 ± 9.1	0.2464
		42	44	6-week HHS, total	89.5 ± 8.1	81.4 ± 9.8	**0.0001**
		35	38	3-month HHS, total	91.2 ± 9.7	91.4 ± 9.7	0.9317
		34	36	6-month HHS, total	95.8 ± 7.8	95.9 ± 6.8	0.968
		34	41	1-year HHS, total	97.5 ± 5.7	97.3 ± 5.5	0.87
		43	44	Pre-op 6MWT	312.3 ± 80.7	291.1 ± 84.5	0.2379
		42	44	6-week 6MWT	513.7 ± 750.5	344.4 ± 96.7	0.1644
		35	38	3-month 6MWT	428.4 ± 95.2	402.3 ± 71.9	0.1842
		42	44	6-week HOOS, symptoms	79.4 ± 12.3	79.9 ± 11.6	0.8631
		35	38	3-month HOOS, symptoms	90 ± 11.5	83.9 ± 11.7	**0.0471**
		34	36	6-month HOOS, symptoms	90.6 ± 12.7	89.7 ± 8.9	0.7404
		34	41	1-year HOOS, symptoms	92.9 ± 13.2	92.1 ± 8.7	0.7574
		42	44	6-week HOOS, pain	83.5 ± 14.7	79.6 ± 16.7	0.2673
		35	38	3-month HOOS, pain	90.8 ± 11.6	89.0 ± 12.5	0.5214
		34	36	6-month HOOS, pain	90.7 ± 14.8	92.6 ± 9.6	0.5288
		34	41	1-year HOOS, pain	94.3 ± 12.7	93.4 ± 10.6	0.7407
		42	44	6-week HOOS, ADL	83.5 ± 13.7	79.0 ± 13.3	0.1341
		35	38	3-month HOOS, ADL	89.1 ± 12.1	89.7 ± 8.6	0.8122
		34	36	6-month HOOS, ADL	92.5 ± 12.7	93.3 ± 7.8	0.7521
		34	41	1-year HOOS, ADL	94.4 ± 11.2	95.4 ± 7.3	0.6518
		42	44	6-week HOOS, QoL	62.6 ± 19.8	54.7 ± 20.5	0.0777
		35	38	3-month HOOS, QoL	76.3 ± 18.2	67.5 ± 19.8	0.0606
		34	36	6-month HOOS, QoL	80.3 ± 20.2	82.3 ± 17.0	0.6615
		34	41	1-year HOOS, QoL	81.3 ± 21.8	85.3 ± 17.5	0.3769
Barrett	2019	41	44	Pre-op UCLA	3.68 ± 1.507	3.07 ± 0.873	**0.026**
		36	39	5-year min UCLA	6.33 ± 1.639	6.26 ± 1.888	0.8516
		42	44	Pre-op HHS	56.7 ± 10.42	53.8 ± 10.19	0.1961
		39	40	5-year min HHS	96.9 ± 8.44	97.1 ± 9.95	0.9417
		39	39	5-year min HOOS Jr	95.7 ± 7.7	92.9 ± 14.1	0.2815
Cao	2020	65	65	Pre-op HHS	45.8 ± 4.0	46.8 ± 6.5	0.272
				1-week HHS	78.7 ± 3.3	71.7 ± 4.1	** < 0.001**
				3-week HHS	84.2 ± 3.4	77.2 ± 3.2	** < 0.001**
				6-week HHS	88.7 ± 2.5	80.0 ± 2.6	** < 0.001**
				3-month HHS	91.6 ± 1.1	91.3 ± 1.3	0.1
				6-month HHS	93.0 ± 1.5	92.9 ± 1.4	0.672
				Pre-op VAS	5.9 ± 1.3	6.2 ± 1.1	0.085
				1-week VAS	2.1 ± 0.7	3.0 ± 0.7	** < 0.001**
				3-week VAS	1.0 ± 0.6	1.7 ± 0.8	** < 0.001**
				6-week VAS	0.5 ± 0.5	0.9 ± 0.8	** < 0.001**
				3-month VAS	0.3 ± 0.5	0.4 ± 0.5	0.599
				6-month VAS	0.2 ± 0.4	0.2 ± 0.4	0.68
Cheng	2017	35	38	Pre-op WOMAC, pain	13.1 ± 3.55	14.6 ± 3.51	–
		35	38	2-week WOMAC, pain	7.5 ± 4.20	7.5 ± 4.19	0.94
		35	37	6-week WOMAC, pain	3.8 ± 3.31	3.7 ± 3.35	0.86
		35	37	12-week WOMAC, pain	1.7 ± 2.72	2.3 ± 2.74	0.33
		35	38	Pre-op WOMAC, stiffness	5.4 ± 1.72	6.1 ± 1.73	–
		35	38	2-week WOMAC, stiffness	3.3 ± 1.95	3.6 ± 1.91	0.64
		35	37	6-week WOMAC, stiffness	2.4 ± 1.66	2 ± 1.64	0.39
		35	37	12-week WOMAC, stiffness	1.4 ± 1.66	1.8 ± 1.64	0.27
		35	38	Pre-op WOMAC, function	44.5 ± 11	50.5 ± 10.97	–
		35	38	2-week WOMAC, function	29.5 ± 12.78	33.4 ± 12.82	0.2
		35	37	6-week WOMAC, function	13 ± 10.53	16.3 ± 10.46	0.2
		35	37	12-week WOMAC, function	6 ± 8.28	8.7 ± 8.27	0.17
		35	38	Pre-op WOMAC, total	63 ± 15.3	71.2 ± 15.29	–
		35	38	2-week WOMAC, total	40.3 ± 17.81	44.5 ± 17.82	0.33
		35	37	6-week WOMAC, total	19.2 ± 14.61	22 ± 14.6	0.43
		35	37	12-week WOMAC, total	9.1 ± 12.13	12.8 ± 12.10	0.2
		35	38	Pre-op OHS	19.1 ± 6.66	14.5 ± 6.66	–
		35	38	2-week OHS	28.5 ± 9.23	26.8 ± 9.25	0.44
		35	37	6-week OHS	39.8 ± 6.21	37.3 ± 6.14	0.1
		35	37	12-week OHS	43.8 ± 5.15	42.8 ± 5.11	0.39
		35	38	Pre-op EQ5D	0.4 ± 0.30	0.3 ± 0.31	–
		35	38	2-week EQ5D	0.6 ± 0.24	0.5 ± 0.25	0.16
		35	37	6-week EQ5D	0.8 ± 0.18	0.8 ± 0.18	0.86
		35	37	12-week EQ5D	0.9 ± 0.12	0.9 ± 0.12	0.57
		35	38	Pre-op EQ5D VAS	61.2 ± 19.4	59.1 ± 19.48	–
		35	38	2-week EQ5D VAS	74 ± 15.97	74.1 ± 15.97	0.98
		35	37	6-week EQ5D VAS	86.6 ± 9.64	87 ± 9.61	0.84
		35	37	12-week EQ5D VAS	91.6 ± 7.75	91.9 ± 7.73	0.87
		35	38	Pre-op 10mWT normal (m/s)	1.1 ± 0.24	1.1 ± 0.25	–
		35	38	2-week 10mWT normal (m/s)	0.9 ± 0.24	0.8 ± 0.25	0.45
		35	37	6-week 10mWTnormal (m/s)	1.2 ± 0.24	1.2 ± 0.24	0.55
		35	37	12-week 10mWT normal (m/s)	1.3 ± 0.18	1.3 ± 0.18	0.85
		35	38	Pre-op 10mWT fast (m/s)	1.5 ± 0.35	1.4 ± 0.37	–
		35	38	2-week 10mWT fast (m/s)	1.1 ± 0.30	1.1 ± 0.31	0.48
		35	37	6-week 10mWT fast (m/s)	1.6 ± 0.24	1.6 ± 0.24	0.9
		35	37	12-week 10mWT fast (m/s)	1.7 ± 0.24	1.7 ± 0.24	0.78
Christensen	2015	28	23	Pre-op chair rising force	48.8 ± 10.8	46.7 ± 8.0	–
				6-week chair rising force	53.2 ± 5.0	50.0 ± 4.8	0.7
				Pre-op TUG	10.3 ± 2.8	12.2 ± 4.4	–
				6-week TUG	8.9 ± 2.5	10.0 ± 2.6	0.51
Moerenhout (Can J Surg)	2020	28	27	Pre-op VAS	5.0 ± 2.4	6.9 ± 2.1	**0.029**
		28	27	2-week VAS	2.0 ± 2.0	2.1 ± 2.0	0.79
		28	27	4-week VAS	1.4 ± 2.0	1.6 ± 1.9	0.63
		28	27	3-month VAS	1.0 ± 1.7	1.1 ± 1.9	0.66
		28	26	6-month VAS	0.4 ± 0.8	0.4 ± 1.0	0.61
		26	24	1-year VAS	0.3 ± 0.5	0.6 ± 1.2	0.38
		26	24	2-year VAS	0.5 ± 0.8	1.0 ± 1.9	1
		28	27	Pre-op HHS	52.1 ± 19.7	48.2 ± 10.1	0.66
		28	27	2-week HHS	66.9 ± 17.1	60.0 ± 15.1	0.12
		28	27	4-week HHS	76.7 ± 16.4	68.7 ± 16.8	0.08
		28	27	3-month HHS	88.4 ± 11.8	83.3 ± 15.1	0.18
		28	26	6-month HHS	90.1 ± 11.3	90.3 ± 12.3	1
		26	24	1-year HHS	94.4 ± 8.0	91.4 ± 13.0	0.72
		26	24	2-year HHS	89.4 ± 11.9	88.7 ± 20.0	0.58
		26	24	5-year HHS	82.0 ± 19.8	80.0 ± 20.4	0.72
Moerenhout (orthopaedics and traumatology)	2021	24	21	Pre-op MHHS	41.7	34.4	0.6
				5-year MHHS	77.5	74.5	0.5
Rykov	2017	23	23	Pre-op HOOS	33.4 ± 16.0	32.5 ± 13.5	0.87
		20	18	6-week HOOS	72.8 ± 16.9	71.0 ± 18.7	0.69
		23	23	Pre-op HHS	52 ± 6.67	51 ± 8.95	0.85
		20	18	6-week HHS	93 ± 10.87	90 ± [9.14	0.36
Taunton	2014	27	27	Pre-op SF12, mental	56.95*	55.73*	0.488
				3-week SF12, mental	58.42*	60.66*	**0.016**
				6-week SF12, mental	58.69*	59.56*	0.262
				1-year SF12, mental	59.84*	57.39*	0.294
				Pre-op SF12, physical	30.28*	34.59*	0.26
				3-week SF12, physical	44.33*	43.45*	0.406
				6-week SF12, physical	53.57*	53.64*	0.4
				1-year SF12, physical	53.80*	53.19*	0.389
				Pre-op WOMAC, pain	45.00*	55.00*	0.051
				3-week WOMAC, pain	97.50*	100.00*	0.294
				6-week WOMAC, pain	100.00*	100.00*	0.111
				1-year WOMAC, pain	100.00*	100.00*	0.364
				Pre-op WOMAC, stiffness	37.50*	50.00*	0.105
				3-week WOMAC, stiffness	75.00*	75.00*	0.101
				6-week WOMAC, stiffness	87.50*	87.50*	0.41
				1-year WOMAC, stiffness	87.50*	87.50*	0.346
				Pre-op WOMAC, function	50.00*	48.53*	0.478
				3-week WOMAC, function	86.76*	91.18*	0.056
				6-week WOMAC, function	97.06*	97.06*	0.392
				1-year WOMAC, function	98.53*	98.53*	0.43
				Pre-op WOMAC, total	47.90*	49.46*	0.202
				3-week WOMAC, total	87.20*	91.49*	**0.043**
				6-week WOMAC, total	95.41*	95.74*	0.287
				1-year WOMAC, total	97.38*	97.38*	0.492
				Pre-op HHS, pain	20*	20*	0.47
				3-week HHS, pain	44*	44*	0.432
				6-week HHS, pain	44*	44*	0.224
				1-year HHS, pain	44*	44/8	0.072
				Pre-op HHS, function	31*	31*	0.476
				3-week HHS, function	37.5*	32*	0.08
				6-week HHS, function	45*	43*	0.079
				1-year HHS, function	45*	44.5*	0.166
				Pre-op HHS, total	55*	51*	0.497
				3-week HHS, total	86.5*	81*	0.085
				6-week HHS, total	97*	93*	0.135
				1-year HHS, total	98*	97.5*	0.231
Taunton	2018	52	49	Post-op VAS	2 ± 1	3 ± 1	** < 0.01**
				Pre-op HHS	57 ± 13	56 ± 12	0.69
				2-month HHS	95 ± 6	92 ± 8	0.07
				1-year HHS	97 ± 4	95 ± 7	0.44
				Pre-op HOOS, symptoms	20 ± 18	16 ± 16	0.35
				2-month HOOS, symptoms	60 ± 12	57 ± 10	0.14
				1-year HOOS, symptoms	69 ± 8	64 ± 13	0.05
				Pre-op HOOS, pain	16 ± 17	16 ± 12	0.98
				2-month HOOS, pain	63 ± 12	61 ± 12	0.54
				1-year HOOS, pain	69 ± 9	67 ± 11	0.41
				Pre-op HOOS, ADLs	20 ± 19	21 ± 15	0.79
				2-month HOOS, ADLs	62 ± 11	61 ± 11	0.61
				1-year HOOS, ADLs	69 ± 10	68 ± 10	0.42
				Pre-op HOOS, sport/recreation	3 ± 24	2 ± 19	0.95
				2-month HOOS, sport/recreation	52 ± 20	51 ± 19	0.94
				1-year HOOS, sport/recreation	63 ± 15	57 ± 17	0.1
				Pre-op HOOS, QoL	-5 ± 16	-1 ± 16	0.21
				2-month HOOS, QoL	49 ± 19	45 ± 19	0.34
				1-year HOOS, QoL	61 ± 18	56 ± 20	0.29
				Pre-op SF 12, physical	30 ± 7	31 ± 7	0.27
				2-month SF 12, physical	45 ± 10	42 ± 8	0.12
				1-year SF 12, physical	49 ± 10	50 ± 7	0.69
				Pre-op SF 12, mental	54 ± 10	53 ± 8	0.91
				2-month SF 12, mental	54 ± 7	55 ± 7	0.65
				1-year SF 12, mental	54 ± 7	54 ± 4	0.82
				Pre-op steps/day	6099 ± 3245	5144 ± 3189	0.23
				2-week steps/day	3897 ± 2258	2235 ± 1688	**0.04**
				8-week steps/day	6665 ± 3247	5503 ± 3523	0.23
				1-year steps/day	6291 ± 3283	5857 ± 3160	0.62
Zhao	2017	60	60	Pre-op pain score	6.12 ± 0.58	6.02 ± 0.43	0.18
				Pre-op VAS	5.95 ± 0.46	5.92 ± 0.67	0.73
				Day 1 VAS	3.07 ± 0.84	3.79 ± 0.96	**0.01**
				Day 2 VAS	2.11 ± 0.28	3.09 ± 0.58	**0.01**
				Day 3 VAS	1.83 ± 0.43	2.49 ± 0.41	**0.01**
				Pre-op HHS	40.19 ± 9.23	43.11 ± 15.59	0.37
				3-month HHS	85.9 ± 17.36	79.6 ± 11.87	**0.04**
				6-month HHS	92.2 ± 13.25	89.9 ± 11.74	0.63
				Pre-op UCLA	4.03 ± 0.29	4.17 ± 0.26	0.22
				3-month UCLA	5.37 ± 1.11	4.12 ± 1.23	**0.03**
				6-month UCLA	7.04 ± 1.13	6.96 ± 1.21	0.67

DAA vs LA		DAA	LA		DAA	LA	
D' Arrigo	2009	20	20	6-week HHS	93.1 ± 7.8	88.3 ± 8	> 0.05
				6-week WOMAC	23.3 ± 9.9	27.7 ± 13.6	**0.003**
De Anta Diaz	2016	50	49	Pre-op HHS	44.4 ± 13.6	42.9 ± 15.2	0.606
				3-month HHS	94.6 ± 10.2	92.8 ± 11.3	0.407
				12-month HHS	96.2 ± 10.1	94.5 ± 9.7	0.397
Dienstknecht	2014	55	88	Pre-op HHS	45.6 ± 15.9	45.6 ± 15.1	0.991
				6-week HHS	78.0 ± 12.7	74.1 ± 13.6	0.142
				3-month HHS	87.1 ± 14.9	85.2 ± 16.5	0.562
				Pre-op OHS	20.0 ± 8.3	19.1 ± 8.0	0.508
				6-week OHS	39.4 ± 7.0	37.0 ± 6.7	0.083
				3-month OHS	41.9 ± 5.4	39.9 ± 8.7	0.196
				Pre-op EQ-5D	0.473 ± 0.235	0.466 ± 0.253	0.859
				6-week EQ-5D	0.847 ± 0.167	0.810 ± 0.169	0.274
				3-month EQ-5D	0.850 ± 0.216	0.845 ± 0.230	0.909
				6 h VAS	1.7 ± 1.7	2.5 ± 2.7	**0.035**
				12 h VAS	1.8 ± 1.9	2.8 ± 2.7	**0.02**
				Day 1 VAS	2.0 ± 1.5	3.4 ± 2.4	** < 0.001**
				Day 2 VAS	2.0 ± 1.9	3.0 ± 2.1	**0.007**
				Day 3 VAS	1.8 ± 1.6	2.7 ± 2.0	**0.01**
				Day 4 VAS	1.7 ± 1.7	2.6 ± 2.0	**0.017**
				Day 5 VAS	1.7 ± 1.7	2.6 ± 2.0	**0.011**
				Day 6 VAS	1.5 ± 1.5	2.2 ± 1.8	**0.03**
				Day 7 VAS	1.5 ± 1.5	2.0 ± 1.7	0.06
				Day 8 VAS	1.4 ± 1.4	1.9 ± 1.6	0.056
Mjaaland	2015	83	80	Pre-op HHS	53.6 ± 13.7	56.0 ± 11.2	-
				Pre-op OHS (0–48)	25.2 ± 7.5	24.8 ± 6.8	-
				Pre-op VAS (0–10)	5.9 ± 1.8	5.7 ± 1.9	-
				Day 1 VAS, before physiotherapy	2.6 ± 2.0	4.0 ± 2.3	** < 0.001**
				Day 1 VAS, after physiotherapy	3.0 ± 2.1	4.6 ± 2.2	** < 0.001**
				Day 2 VAS, before physiotherapy	1.9 ± 1.8	3.0 ± 2.3	**0.001**
				Day 2 VAS, after physiotherapy	2.0 ± 1.8	3.6 ± 2.2	** < 0.001**
				Day 3 VAS, before physiotherapy	1.6 ± 1.7	2.8 ± 2.1	** < 0.001**
				Day 3 VAS, after physiotherapy	1.9 ± 1.9	3.1 ± 2.1	** < 0.001**
				Day 4 VAS, before physiotherapy	1.5 ± 1.7	2.3 ± 1.9	**0.006**
				Day 4 VAS, after physiotherapy	1.8 ± 1.8	2.9 ± 1.9	** < 0.001**
Mjaaland	2019	83	80	3-month OHS	39 ± 7	36 ± 7	**0.02**
				12-month EQ-5D index	0.83 ± 0.18	0.77 ± 0.20	**0.04**
Nistor	2020	56	56	After passive PT (day 1) VAS	2*	4*	** < 0.001**
		56	56	After active PT (day 2) VAS	2*	4*	** < 0.001**
		56	56	After active PT (day 3) VAS	2*	3*	** < 0.001**
		56	56	After active PT (day 4) VAS	2*	3*	** < 0.001**
		54	55	After 20mWT (6 week) VAS	1*	1*	**0.009**
		54	53	After 20mWT (3 month) VAS	0*	1*	0.062
		48	47	After 20mWT (6 month) VAS	0*	0*	0.293
		40	39	After 20mWT (1 year) VAS	0*	0*	0.424
Reichert	2018	77	71	Pre-op HHS	54.0 ± 14.2	53.0 ± 15.7	0.2813
		76	53	6-week HHS	81.6 ± 12.1	82.4 ± 12.0	0.068
		75	53	3-month HHS	89.8 ± 9.3	88.4 ± 9.9	0.37
		75	50	6-month HHS	90.3 ± 9.8	89.1 ± 10.0	0.556
		73	50	12-month HHS	92.4 ± 8.6	91.4 ± 9.1	0.477
		77	71	Pre-op XSFMA, function	35.2 ± 16.1	40.5 ± 16.0	0.053
		76	53	6-week XSFMA, function	21.2 ± 14.2	28.5 ± 15.9	**0.026**
		75	53	3-month XSFMA, function	12.7 ± 12.5	18.8 ± 16.1	**0.023**
		75	50	6-month XSFMA, function	11.6 ± 12.1	15.8 ± 15.4	0.094
		73	50	12-month XSFMA, function	10.3 ± 13.0)	15.1 ± 16.3	**0.04**
		77	71	Pre-op XSFMA, bother	48.7 ± 20.5	53.0 ± 17.9	0.126
		76	53	6-week XSFMA, bother	26.6 ± 19.8	33.0 ± 18.3	0.055
		75	53	3-month XSFMA, bother	19.8 ± 17.0	33.0 ± 18.1	0.099
		75	50	6-month XSFMA, bother	16.8 ± 15.8	25.1 ± 17.9	0.149
		73	50	12-month XSFMA, bother	15.8 ± 18.0	21.7 ± 19.6	0.056
		77	71	Pre-op SF36, physical	27.4 ± 8.2	25.6 ± 8.7	0.152
		76	53	6-week SF36, physical	39.1 ± 9.7	34.8 ± 9.8	**0.004**
		75	53	3-month SF36, physical	44.6 ± 9.2	40.7 ± 10 1	**0.031**
		75	50	6-month SF36, physical	46.0 ± 10.0	42.7 ± 5.6	**0.042**
		73	50	12-month SF36, physical	47.5 ± 9.9	42.9 ± 11.9	**0.017**
		77	71	Pre-op SF36, mental	57.2 ± 8.5	56.3 ± 9.2	0.405
		76	53	6-week SF36, mental	58.1 ± 8.7	59.3 ± 66	0.465
		75	53	3-month SF36, mental	56.0 ± 9.2	56.7 ± 8.3	0.774
		75	50	6-month SF36, mental	56.0 ± 10.0	55.8 ± 72	0.67
		73	50	12-month SF36, mental	55.0 ± 9.8	56.2 ± 6.9	0.714
		77	71	Pre-op Stepwatch Activity Monitor	4695	4695	-
		75	53	3-month Stepwatch Activity Monitor	5992	5239	**0.035**
		73	50	12-month Stepwatch Activity Monitor	6402	5340	**0.012**
		77	71	Pre-op T25-FW (s)	22.4 ± 5.2	24.0 ± 3.9	0.193
		76	53	6-week T25-FW (s)	21.3 ± 6.3	22.0 ± 4.2	0.385
		75	53	3-month T25-FW (s)	18.5 ± 3.7	19.4 ± 3.8	0.291
		75	50	6-month T25-FW (s)	18.3 ± 4.1	19.9 ± 5.5	**0.04**
		73	50	12-month T25-FW (s)	18.1 ± 3.4	19.8 ± 4.6	**0.046**
		77	71	Pre-op activity VAS	5.0 ± 0.8	4.9 ± 0.8	0.461
		76	53	6-week activity VAS	6.9 ± 0.7	6.8 ± 0.6	**0.031**
		75	53	3-month activity VAS	7.3 ± 0.8	6.9 ± 0.5	0.08
		75	50	6-month activity VAS	7.3 ± 0.7	6.9 ± 0.7	0.223
		73	50	12-month activity VAS	7.5 ± 0.6	7.0 ± 0.7	** < 0.001**
		73	50	12-month walking distance (m)	6435 ± 4260	5125 ± 3868	**0.045**
Restrepo	2010	50	50	Pre-op HHS	51.86	54.95	0.06
				6-week HHS	93.64	88.8	**0.03**
				6-month HHS	94.45	90.03	**0**
				1-year HHS	94.72	92.08	**0.04**
				2-year HHS	97.34	97.55	0.72
				Pre-op LEFS	6.72	6.51	0.25
				6-week LEFS	10.36	9.9	0.36
				6-month LEFS	10.12	9.56	**0.04**
				1-year LEFS	10.3	10.12	0.5
				2-year LEFS	10.58	10.14	0.07
				Pre-op WOMAC	8.68	8.33	0.29
				6-week WOMAC	4.4	9.7	**0**
				6-month WOMAC	3.46	8.62	**0**
				1-year WOMAC	3.68	6.06	**0.02**
				2-year WOMAC	2.24	1.9	0.6
				Pre-op Linear Analogue Scale, Energy	5.89	5.72	39
				6-week Linear Analogue Scale, Energy	7.71	7.15	0.06
				6-month Linear Analogue Scale, Energy	7.82	7.29	0.06
				1-year Linear Analogue Scale, Energy	7.9	7.43	0.11
				2-year Linear Analogue Scale, Energy	7.96	7.91	0.63
				Pre-op Linear Analogue Scale, Daily Activity	6.6	6.46	0.36
				6-week Linear Analogue Scale, Daily Activity	8.13	7.48	0.49
				6-month Linear Analogue Scale, Daily Activity	8.29	7.84	0.19
				1-year Linear Analogue Scale, Daily Activity	8.35	7.91	0.19
				2-year Linear Analogue Scale, Daily Activity	8.08	8.14	0.57
				Pre-op Linear Analogue Scale, Overall	6.07	5.93	0.57
				6-week Linear Analogue Scale, Overall	8.23	7.33	**0**
				6-month Linear Analogue Scale, Overall	8.54	7.75	**0.02**
				1-year Linear Analogue Scale, Overall	8.59	7.79	**0.01**
				2-year Linear Analogue Scale, Overall	8.23	8.26	0.88
				Pre-op SF36, Physical	68.91	66.32	0.27
				6-week SF36, Physical	87.74	70.35	**0**
				6-month SF36, Physical	89.02	75.14	**0**
				1-year SF36, Physical	89.22	84.78	0.13
				2-year SF36, Physical	90.44	91.11	0.6
				Pre-op SF36, Mental	26.86	28.98	0.57
				6-week SF36, Mental	89.7	81.3	**0**
				6-month SF36, Mental	90.64	79.72	**0**
				1-year SF36, Mental	90.16	86.85	0.18
				2-year SF36, Mental	92.51	92.9	0.58
Zomar	2018	36	42	Pre-op WOMAC, pain	48.89 ± 15.9	44.02 ± 16.85	0.2
		36	41	6-week WOMAC, pain	73.21 ± 14.22	76.65 ± 14.02	0.29
		33	40	12-week WOMAC, pain	83.65 ± 12.47	89.16 ± 12.33	0.06
		36	42	Pre-op WOMAC, stiffness	43.40 ± 20.58	42.99 ± 17.24	0.92
		36	41	6-week WOMAC, stiffness	64.27 ± 16.56	69.22 ± 16.39	0.19
		33	40	12-week WOMAC, stiffness	74.67 ± 14.99	73.97 ± 14.86	0.84
		36	42	Pre-op WOMAC, function	47.10 ± 16.56	42.50 ± 13.67	0.18
		36	41	6-week WOMAC, function	73.44 ± 14.7	74.72 ± 14.54	0.71
		33	40	12-week WOMAC, function	82.48 ± 12.64	84.82 ± 12.52	0.43
		36	42	Pre-op WOMAC, total	47.07 ± 16.32	43.24 ± 12.83	0.27
		36	41	6-week WOMAC, total	71.50 ± 13.26	74.30 ± 13.06	0.36
		33	40	12-week WOMAC, total	81.34 ± 11.60	84.35 ± 11.5	0.27
		36	42	Pre-op SF12, physical	33.19 ± 9.72	31.04 ± 6.93	0.26
		36	41	2-week SF12, physical	31.05 ± 7.8	30.37 ± 7.75	0.71
		36	41	6-week SF12, physical	40.65 ± 9.24	40.68 ± 9.16	0.99
		33	40	12-week SF12, physical	45.92 ± 8.21	46.67 ± 8.10	0.7
		36	42	Pre-op SF12, mental	55.57 ± 12	51.43 ± 11.21	0.12
		36	41	2-week SF12, mental	52.52 ± 10.14	54.09 ± 9.99	0.5
		36	41	6-week SF12, mental	52.80 ± 9.54	54.07 ± 9.41	0.56
		33	40	12-week SF12, mental	55.16 ± 8.10	55.81 ± 7.97	0.73
		36	42	Pre-op HHS	63.16 ± 8.34	58.04 ± 11.99	**0.04**
		33	40	12-week HHS	95.44 ± 7.18	92.04 ± 7.08	0.05
		36	42	Pre-op VAS	5.32 ± 2.4	6.24 ± 1.75	0.06
		36	41	DC VAS	4.17 ± 2.64	3.86 ± 2.50	0.66
		36	41	2-week VAS	2.76 ± 2.28	2.74 ± 2.24	0.98
		36	41	6-week VAS	1.57 ± 1.92	1.04 ± 1.86	0.23
		33	40	12-week VAS	0.85 ± 1.67	0.60 ± 1.64	0.52

*HHS* Harris Hip Score, *MHHS* : Modified Harris Hip Score, *OHS* :Oxford Hip Score, *WOMAC*  Western Ontario and McMaster Universities Osteoarthritis Index Score, *EQ-5D*   EuroQoL 5-Dimension, *HOOS*   Hip Disability and Osteoarthritis Outcome Score, *VAS*  Visual Analogue Scale, *SF12*   12-Item Short Form Health Survey, *SF36*   36-Item Short Form Health Survey, *UCLA*   University of California Los Angeles activity scores, *LEFS*  Lower Extremity Functional Scale, *TUG*  timed up and go, *XSFMA*  extra short musculoskeletal functional assessment, *mWT* meter walk test, *MWT* minute walk test, *T25-FW* timed 25-m foot walk. *median values presented. Bolded *p*-values are meant to highlight statistical significance
